# More Than Just Assistive Devices: How a South African Social Enterprise Supports an Environment of Inclusion

**DOI:** 10.3390/ijerph18052655

**Published:** 2021-03-06

**Authors:** Zara Trafford, Erna van der Westhuizen, Shona McDonald, Margi Linegar, Leslie Swartz

**Affiliations:** 1 Psychology Department, Stellenbosch University, Stellenbosch 7602, South Africa; lswartz@sun.ac.za; 2 Shonaquip Social Enterprise, Cape Town 7800, South Africa; erna@uhambofoundation.org.za (E.v.d.W.); shona@shonaquip.co.za (S.M.); margi@linegar.co.za (M.L.)

**Keywords:** childhood disability, assistive devices, inclusion, participation, low-resource settings, South Africa

## Abstract

Appropriate assistive technology has the potential to considerably enhance quality of life, access to health and education, and social and economic participation for people with disabilities. Most disabled people in the world live in low- and middle-income countries where access to assistive devices and other support is severely lacking. There is little evidence that describes contextually relevant approaches to meeting these needs, particularly in African countries. We provide a detailed description of a South African organisation which has manufactured mobility and seating devices for children with disabilities since 1992. The Shonaquip Social Enterprise (SSE) also trains and builds capacity among a wide range of stakeholders (caregivers, health workers, educators, government, and communities) to acknowledge and advocate for the wellbeing of disabled children and adults, and works closely with government to strengthen existing service provisions. Using examples from the SSE, we highlight a number of useful principles to consider when trying to provide for the needs of people with disabilities, particularly in low-resource settings. While access to assistive devices is important, devices have limited capacity to improve participation if the broader environment is overly restrictive and stigmatising. Improved access to devices ought to be situated within a range of broader efforts to increase the inclusion and participation of people with disabilities.

## 1. Introduction

Globally, the majority of people with disabilities live in low- and middle-income countries (LMICs) [1,2]. Supports including personal assistance and appropriate assistive technology have the potential to considerably improve quality of life, access to education, and social and economic participation for disabled people [3,4]. However, the World Health Organization (WHO) estimates that only about 10% of people who would benefit from appropriate assistive devices are currently able to gain access [5]. Adequate and properly trained personal assistance is also difficult to obtain in settings where financial and human resources are scarce. In many LMICs, barriers to access may include a lack of dedicated resources (because disability is still absent from many social justice conversations), as well as dominant negative attitudes and widespread stigma about disability [6,7]. The compounding effects of high levels of poverty and rurality can create particularly impenetrable barriers for people with disabilities [8,9]. Additionally, there is a lack of rigorous and contextually relevant evidence about people’s need and methods for delivering appropriate and cost-effective assistive devices and technology [10], especially in African settings [4,11].

In terms of disability-related legislation, policy, and targeted government assistance, South Africa is in a better position than many LMICs [9]. Both the national Constitution and the United Nations Convention on the Rights of Persons with Disabilities (UNCRPD), on which South Africa has been a signatory since 2007, theoretically enshrine inclusion and protection for people with disabilities. There are also a number of specific commitments that have been made to upholding the rights of people with disabilities. For example, disabled people have the right to inclusion in schooling and there is a movement toward mainstreaming; have the right to access health services for free and without prejudice (although this is a right for all residents not only disabled people); and are included in employment equity and supposedly protected from workplace discrimination [12,13,14,15]. Deductions can be claimed on tax returns for some disability-related costs but this is not relevant for a large proportion of the population, as the country’s tax-paying base is small. In 2019, only 31% of 21.1 million tax-registered individuals (approximately 6.5 million people) earned enough to complete a tax return, in a population of 58 million [16]. There are also government-funded schemes available to compensate for workplace accidents (Compensation for Occupational Injuries and Diseases Fund) and for disability caused by road accidents (Road Accident Fund) [17,18]. However, as is the case with much other social programming, implementation has been slow, inconsistent, and inadequate [8,14,15]. There is also a lack of synergy between different government departments and their agendas, despite the fact that disability can only be addressed intersectorally. Many ideological commitments to people with disabilities have not yet been translated into law or practicable guidelines [17,19]. For example, although government’s commitments to disabled people were reaffirmed in the 2016 White Paper on the Rights of People with Disabilities [20], by 2020 the Department of Social Development had not yet promulgated any disability-specific legislation in line with the White Paper [14]. Depending on which of South Africa’s nine provinces is examined, each province’s relative resources, organisational capacity, and guidelines cause further variations in access. Another background factor is poor national capacity to properly identify people with disabilities and their related needs, whether for linkage to care, or in order to assess disability for social protection benefits [17,21].

In terms of government-funded social protection for people with disabilities, children may be recipients of the care dependency grant (CDG), a cash transfer grant which amounted to ZAR1,860.00 (USD125.25) in 2020, that is provided to the primary caregiver of a child with a disability in need of permanent care [17]. This grant is contingent on a doctor’s assessment, which uses a framework based in the now outdated medical model of disability [22,23]. Attempts have been made to update these assessment tools but have been stalled by challenges with intersectoral work (the Departments of Social Development and Health have to collaborate in the process) and a lack of clarity about “ownership” of the issue in existing legal guidelines [24]. For people living far from health services, visiting a doctor for an assessment can be expensive and time-consuming in terms of travel costs and waiting times. It is common for people accessing primary care services in overburdened or under-resourced areas to be turned away at the end of a full day of waiting and asked to return the next day [25,26]. The administrative aspects of the process can thus be slow and inconsistent. The CDG is under-subscribed and supports the least recipients of all social grants in the country [17]. While the prevalence of childhood disability is not easy to assess [17], the current tools used for estimating disability in South Africa are considered inappropriate by local researchers and disability rights organisations [24,27]. Children who are living in poverty (and may have an impairment but do not qualify for or cannot access the CDG) may be beneficiaries of the child support grant (CSG), a smaller grant with the highest number of beneficiaries of all social grants [8]. For adults, there is a disability grant (DG) available for income replacement, which is contingent on an assessment of ability to work. This is one of the most highly subscribed grants in the country but it is quite controversial [17,22,23,28].

Research has indicated that social grants are crucial for the survival of poorer households, they are still insufficient for properly and sustainably moving people out of poverty [29,30,31]. Disability grants are around four times the amount of poverty-alleviation grants, but for most recipients, disability grants still do not meet their needs because of the very high costs associated with disability [8,32]. Care dependency grants are expected to cover a wide range of expenses that could include one or more of the following: full-time care (either from a carer or by “buying out” the parent or primary caregiver’s time), education, specialised food, diapers, as well as the purchase and maintenance of assistive devices [33]. Should a visit to an emergency room be needed, for example, half of the month’s grant could be spent arranging a private taxi to take the child to hospital, as there are insufficient ambulances available and most public transport is not accessible for people with mobility impairments [32,34].

Next, we highlight how some gaps between principle and practice manifest in the health and other sectors, specifically with regard to people with mobility impairments, an example relevant to this paper and the theme of this Special Issue. The public sector health system serves approximately 84% of the population but the vast majority of human and other resources for health are concentrated in the country’s private sector [35]. For those who cannot afford to access care in the private sector, the provision of mobility devices is administered by the public sector [36]. Broader inadequacies in the health system are thus also reflected in the area of delivery of assistive devices and rehabilitation support, and are intensified by the proven lack of access to transport and lower income associated with disability in South Africa [8,14,32,37,38]. There is an overall shortage of rehabilitation and occupational therapists, a lack of emphasis on community-based rehabilitation (CBR) approaches, and insufficient training in the prescription, adjustment and maintenance of assistive devices [39,40,41], all of which results in a mismatch between legal entitlements and reality. The 2016 Framework and Strategy for Disability and Rehabilitation Services (FSDR) confirmed that comprehensive rehabilitation services should be available at all levels of care, and ought to be founded on CBR principles [42]. Once again, by 2020 no provisioning had been made to deliver on this vision, and there are still no provincial implementation guidelines [42]. The South African National Guidelines on Provision of Assistive Devices purportedly guarantee access for all those who need them [43]. However, while a physically disabled person may be technically entitled to a wheelchair, a severe lack of devices and overly long waiting lists means that this right is often not actualised, especially for those living in poorer or rural areas. Women have reported carrying their disabled children on their backs (as is customary for carrying infants) up until the age of 13 [32]. Therapists are sometimes forced to improvise mobility and seating devices by using cardboard or homemade wooden supports [36]. When access is gained, the wheelchair may have been repaired multiple times, or may still be broken on receipt [32,34]. This is especially the case for people who are likely to live in already challenging environmental contexts, such as communities where access to electricity, sanitation, and safe housing is non-existent or unreliable. Despite these constraints, many health workers are enterprising and often go far beyond the call of duty or use their own personal resources to address such gaps. However, this situation is inadequate and unsustainable and despite substantial legal entitlements, provisions for most disabled children and adults fall far short of meeting recommended standards.

For children with mobility impairments, seating and related assistive devices can facilitate supported or independent movement, prevent infection and disease, and assist in managing or minimising painful effects of the existing disability [4,44,45,46,47,48]. For example, the right kind of seating device can prevent additional spine curvature in children with certain kinds of physical impairment [49]. Such devices also play a critical role in facilitating disabled children’s access to and engagement in educational, social, and other public spaces, improving the likelihood of their inclusion and social participation [3,50]. However, accessing these devices can be financially prohibitive [51], and “availability,… adaptability, acceptability and quality” are also critical considerations in delivering these services [36,50]. The effective delivery of contextually relevant devices and technology must also be supported by professionals who can appropriately prescribe, adjust, and maintain these devices, as well as train and support both the user and their parent or caregiver [36,44,52]. As noted above, this is still lacking in the South African context. There is also a growing understanding that the exclusion of disabled people both increases their own economic vulnerability and is also societally costly [53]. However, without parallel work with caregivers, schools, and the broader social and economic community, assistive devices and technology have limited capacity to improve participation. Reaching the end goal of fuller inclusion and participation means that access to devices ought to be one of a range of broader, holistic efforts to improve the acknowledgement of and provisions for people with disabilities by government and society [54]. Useful approaches should include innovation in community- and capacity-building, disability rights awareness raising, and political advocacy toward the empowerment of people with disabilities [55]. Additionally, this will allow those who currently do not have a disability to meet and learn from a more diverse range of people, which will be mutually beneficial.

In this article we consider the approach of a South African social enterprise doing innovative work in the field of assistive devices for children in South Africa and further afield. The social enterprise brings together the diverse but linked threads of device provision, capacity-building, and activism required to achieve more comprehensive, sustainable change. While the focus of their device production is on children with mobility and mobility-related impairments, their work has expanded over time and those impacted now also include children with other disabilities, adults with disabilities, and disabled people’s networks of families, educators, and health and social workers. The organisation also works closely with government in numerous ways to strengthen existing public provisions. This paper is not based on a formal evaluation or a scientific research project. Instead, we present a reflection on practice based on the perspectives of a mixed authorship group. We centre the “insider” perspectives of co-authors who are also staff of this social enterprise and pair this with insights from two academic co-authors involved in disability research. We wish to be clear at the outset that our approach here cannot in any way be considered an evaluation of the work of the organisation. From an emancipatory disability studies perspective, however, it is important that academic texts on disability include a wider range of voices, especially those with lived experience of the issues under discussion [56]. In this case, these voices include those of the organisations working to promote disability inclusion. We hope through this article to generate discussion and debate on improving access to devices, social inclusion, and disability rights in South Africa and more broadly.

## 2. A South African Example: The Shonaquip Social Enterprise

The Shonaquip Social Enterprise (SSE) provides an illustrative example of a broad-based approach to facilitating social participation and improved quality of life for people with disabilities in South Africa. The SSE works to increase access to appropriate devices, as well as to build technical and clinical capacity among rehabilitation professionals and educators nationwide. The SSE also aims to strengthen referral pathways for people with disabilities, provides support and network-building for caregivers and families, offers a range of outreach and mentoring programmes, and conducts community sensitisation and disability rights awareness events. Rather than replacing government services, the SSE builds sustainability by collaborating with government and other local service providers to enhance and improve the quality and consistency of services. The SSE has a specific interest in reaching people in areas that are far removed from rehabilitation services and medical care. Here, the support and services people are legally entitled are particularly likely to be fragmented, inconsistent, or not well-publicised.

The social enterprise’s work is organised according to their “ecosystems for inclusion” framework, which is strongly influenced by the social model of disability and a rights-based approach to improving disabled people’s participation in society. The SSE comprises Shonaquip, the Uhambo Foundation, and a range of other activities and services where these entities overlap and programming is delivered jointly. Through partnerships and collaboration, the SSE tries to deliver or facilitate service provision and advocacy in four interlinking and overlapping areas: (1) barriers, perceptions, and local referrals, (2) posture support and seating services, (3) education, and (4) learning and economic participation. These are considered the primary areas in which intervention and progress must happen in order for wider systemic change to occur. The SSE’s activities have a wide reach, as described in its 2019 impact report. For example, in that year for category 2 above (postural and support/seating activities), 1520 devices were provided in South Africa and 470 in other African countries. 254 product training events took place, targeted at families, carers, and therapists. 228 therapists and 431 clients were reached by outreach activities. The SSE also provided refurbish and repair services to 140 devices [57].

Since its inception, the SSE has worked in different capacities with government service providers and decision-makers. Shonaquip initiated this relationship with advocacy, and played a strong role in motivating for the development of the government’s assistive devices policy. Earliest collaborations were with government-employed therapists, who provided input on the design of chairs and postural supports for children. Over the years, the social enterprise has worked with government as a capacity-builder and trainer, supplier, and implementer. SSE also plays a strong government monitoring and accountability role, having represented and advocated for the interests of their beneficiaries and other disabled South Africans for many years. SSE has made and continues to make regular contributions to the design and review of South African disability and assistive device policy. Government also acts as both purchaser and recipient of devices, as detailed further below. In a different role, the SSE has trained government service providers, device technicians, clinicians, and community workers. For example, SSE partnered with the national Department of Health in the design of its current basic wheelchair course, which provides local seating practitioners with WHO-aligned training in wheelchair service provision. SSE was later subcontracted by government to deliver ongoing outreach clinics in one region in the Western Cape province. Also in the Western Cape, the SSE helped to conduct and improve a large provincial household survey. Both remote and periodic in-person mentoring helps to further enhance the services offered in other parts of the country [58].

The SSE uses a hybrid social enterprise model that sources funding for much of its work from a combination of funds raised through the manufacturing and distribution of its support devices, as well as funded programmes for community impact [59]. 60% of Shonaquip’s devices are purchased by government tenders, for distribution in the public health and education sectors. A further significant proportion are purchased through public–private partnerships (PPPs) initiated by the SSE. PPPs enable the funding of devices for children on existing public sector waiting lists—once funded, these devices are donated to public sector health services (at either district or provincial hospitals). These PPPs are usually funded by corporate sponsors or South African development and human rights funders. SSE’s rural outreach programmes also assist with the identification and wait-listing of individuals not able to access state services. In future, the SSE aims to approach more international funders to fund scaled-up programming for further regional expansion. Local non-governmental organisations (NGOs) account for a further 30% of device purchasing, on behalf of their beneficiaries. These NGOs range from international NGOs (INGOs) to small centres, and are generally funded by grant-makers or through other fundraising efforts. Private sales contribute a further small proportion of SSE funding. A final source of operational funding is the payment SSE receives for providing disability-related training and services to the Departments of Health, Education, and Social Development, as well as directly with community stakeholders. This emphasis on strengthening and facilitating government service and support provisions is aimed at building capacity to encourage and facilitate systemic improvements. Providing adaptable, context-appropriate devices for government distribution strengthens existing service delivery. Shonaquip devices are high-quality but also significantly more affordable than local alternatives, which increases sustainability and helps limited funds reach more people. Partnership with government is a key strategic decision. If government systems can identify, link relevant people to care, and retain them within the system, they are more likely to receive age-appropriate services. Contact with government services also has a positive effect on disability data. If children (and adults) with disabilities can be identified in the public sector, they are more likely to be counted and more likely to have provisions made for their needs when policies and budgets are planned, a sentiment also expressed in 2017 by the South African Human Rights Commission [21].

Below, we provide a detailed description of the establishment, expansion, and impact of this organisation. We then link the SSE’s work to a number of existing best practice recommendations for the provision and delivery of support services and assistive devices in low-resource settings, with the end goal of proper inclusion of children with disabilities—for their benefit as well as the benefit of their peers and society at large. We do not suggest that this is the only way of meeting such needs or providing support, but believe that the long-term and embedded nature of the SSE’s work offers rich ground for reflection that may generate interesting debate.

### 2.1. Shonaquip

Shonaquip produces mobility and seating devices for children with mobility and other impairments and is one part of the Shonaquip Social Enterprise. Shonaquip was founded in Cape Town, South Africa in 1992 by Shona McDonald, whose second daughter was born with cerebral palsy. McDonald realised that the range of devices available at the time in South Africa was wholly insufficient, especially for children with considerable difficulty in maintaining posture (i.e., the inability to sit upright or balance without substantial postural support). Children with mobility impairments would usually be strapped into a standard pram with a cardboard insert to support their posture, which did not truly facilitate mobility and could even worsen physical wellbeing by increasing the curvature of the spine or causing avoidable pressure sores. Even for those who could access better wheelchairs, the chairs available were usually designed in a wealthy country with better physical infrastructure and were thus inappropriate for many local terrains and environments. In much of South Africa, people live in rural areas with uneven surfaces and commonly, thorns and stones in the road. Even in urban and peri-urban settings, low-resourced wards (a ward is a geopolitical subdivision within a South African municipality) and municipalities struggle to maintain the quality of roads and pavements. 

McDonald was an artist with a background in sculpting and wanted to help solve this local problem. She initially collaborated with a biomedical engineer from the University of Cape Town, as well as a number of rehabilitation specialists, who contributed to the development of seating devices and attendant training processes. At that stage the group were focused on designing high quality seating that included proper postural support, but that could also be customised and adapted for the specific needs of each user. Shonaquip has since established a reputation for producing sturdy, supportive seating and mobility devices for individuals across the country. They have won design awards for their collaborative approach and the therapeutic value of their devices [60]. As detailed above, Shonaquip has also partnered with government to extend service provision for people with disabilities and to build the country’s capacity to deliver comprehensive wheelchair services, in line with WHO standards [61]. The majority of Shonaquip’s device production and related wheelchair services work is in South Africa’s nine provinces but they have also extended regional distribution to a few nearby countries (Botswana, Mozambique, Namibia, Zambia, Zimbabwe), as well as to Georgia, India, Iraq, Tanzania, Uganda, the United States of America, and the United Kingdom.

More recently, Shonaquip has supported the South African government’s efforts to address disability awareness and facilitate inclusion. The social enterprise also advocates more broadly for the economic inclusion of people with disabilities, and offers an advisory service for local companies seeking to improve disability inclusion in their workforce. 30% of the Shonaquip staff is disabled, indicating their commitment to and role-modelling of the economic inclusion of people with disabilities. Many of these employees require a device themselves and understand first-hand how the quality of the device can impact on the participation potential of the user. Their insights are incorporated into the organisation for design and awareness raising. For example, in one instance interviews were conducted with factory staff who were also wheelchair users. Their stories were then used to inform and educate other colleagues and the organisation at large.

Various mobility and seating supports are now produced to meet different needs. These include a diverse range of manual or powered buggies that work on a modular and customisable system, as well as removable positioning supports for standing, sitting, lying down, and traveling in a vehicle. These devices are aligned with WHO and other best practice recommendations [61,62] but their design is also strongly influenced by insights from users and their caregivers, as well as by an understanding of common local environments. For example, the original Shonaquip MadibaBuggy was developed in the 1990s to address the abovementioned gap in available provisions. The original MadibaBuggy was intended specifically for children who could not self-propel or were unable to sit independently and needed considerable posture support. The buggy also assisted children with the control of spasms and muscle imbalances. Later, with the help of new industrial design staff, the chair was updated and enhanced to create the Madiba2GoBuggy. The latter still centres the needs of the user but also incorporates the needs of the caregiver who works with the chair every day, and the therapist who adjusts the seating. The Madiba2GoBuggy is especially suited to rugged terrain (roads and informal paths which are often gravelly, sandy, or muddy, potholed, and hilly), can recline, and has a number of other adjustable features to allow for easier transport, storage, and handling by the carer. Unlike standard wheelchairs, both buggies have wide off-road wheels and frames that are sturdier than usual to handle uneven terrain. These frames are also designed to be easy to maintain and repair in under-resourced or distant areas where specialist shops or technicians are unavailable. Most recently, in light of the difficulty of transporting chairs across the long distances between provinces and from urban centres to rural outskirts, Shonaquip has worked on producing deconstructed “flat pack” wheelchairs, which allows for wider distribution and also provides employment opportunities for people who can be trained to assemble these products on-site.

In response to the aforementioned shortage of appropriately trained providers who can prescribe and adjust assistive devices, Shonaquip expanded their work and moved beyond the design, manufacturing, and distribution of seating and mobility devices. Shonaquip now provides services for the initial assessment, customisation, fitting, and maintenance of devices. To expand on this impact, they also train healthcare professionals in the maintenance, prescription, and adjustment of relevant assistive technology. Along with assessing the physical and cognitive needs and capacity of the user, the comprehensive assessment training process emphasises a wide range of important considerations. These include the user’s home background and lifestyle (the activities that are most important to the user or that they hope to pursue in the future), accessibility in their home and specific neighbourhood, their access to public or private transport, and if any other support is available [63].

### 2.2. The SSE’s Community-Based Programming—The Uhambo Foundation; Training, Outreach, and Linkage to Care; and Caregiver Empowerment

After years of impactful device and assessment service provision, Shonaquip realised that a more holistic approach was needed and decided to expand their efforts into community-based programming. While an appropriate assistive device is important as a tool for enabling participation, it was understood that a device on its own could not address a range of other barriers that prevent children’s inclusion and full participation. In expanding, the SSE aimed to work closely with caregivers of children with disabilities, to capacitate educators of children with disabilities, and to raise broader societal awareness about and advocate for the inclusion of people with disabilities in South Africa. Established in 2010, the Uhambo Foundation implements and support Shonaquip service provision by means of a range of community outreach and capacitation initiatives. A number of other activities are also jointly delivered, under the umbrella of the Shonaquip Social Enterprise and their ecosystems approach.

Currently working in most South African provinces and intending to expand their work, the SSE has launched a range of programmes including “Let’s Talk” dialogues (Uhambo Foundation community-based disability awareness raising events), family support, educator and caregiver skills development, and policy advocacy. The SSE aims to be responsive and context-aware. In low-resourced areas in South Africa, as in many other LMICs, health and other community-based social development programmes regularly emerge but often disappear as soon as the funding concludes, or if the programme is not locally acceptable [44]. Such programmes are also vulnerable to high staff turnover and lack of appropriate handover to local implementers, especially in rural areas. Although the SSE does offer specific services and resources, they prioritise enhancing *existing* local services to avoid wastage, work duplication, and a dependency on external partners, which often make this kind of work unsustainable. They work to strengthen services, bolster referral systems, identify community barriers and ways to address them, and facilitate connections between people who may be able to support or help each other but are not yet connected. They also upskill existing social and development workers to better understand and champion the rights of people with disabilities and to provide them with useful resources, such as standardised referral forms that encourage linkage to relevant service providers or clinicians. For example, although a cadre of social workers may already be working in a particular area, they may not yet have the specific skills or networks to properly cater for disabled people. The SSE might then identify these individuals, provide targeted training, and facilitate connections between these social workers and nearby caregivers who may feel isolated.

Additional capacity-building efforts have included support for a range of outreach “clinic” services run by a seating practitioner and a wheelchair technician. These services provide specialised training and mentoring in clinical and technical skills for existing local providers and community workers. The clinics are hosted in collaboration with local community-based sites including primary health care clinics, schools, institutions and care centres. In so doing, local hosts are equipped and empowered to actively expand on their service provision by consciously including disabled people. The ultimate goal is for these clinics eventually to be completely independent. In other words, the SSE tries to “do with and alongside” rather than “do for”. In South Africa, much of the limited but important existing rehabilitation therapy workforce includes newly graduated occupational therapists and physiotherapists, who are then often appointed in remote areas to provide services during their one year of compulsory community service. The SSE offers inputs for these therapists during their university training years to prepare them for the challenges they may find in the field. These varied and contextually relevant approaches to capacity-building increase the chance that children with disabilities in the region will receive an appropriate device assessment and prescription. Further representing this adaptive approach to service delivery, during the coronavirus pandemic the organisation’s training had to shift toward more flexible designs and alternative platforms to replace hands-on training. For example, the design of a range of methods for instruction such as training videos and “frequently asked questions” lists, which are shared on the organisation’s website. They have also used more innovative approaches to content delivery and networking, like using social media platforms for webinars and instant messaging groups to connect parents, carers, teachers, and other stakeholders.

Another example of the SSE’s sustainable approach can be found in their early childhood development (ECD) work. In South Africa, there are local ECD centres (i.e., pre-school/creche facilities) in many low-resource or rural areas. ECD centres are often run by local women to provide care for young children in under-served areas and to generate an income. Obtaining a basic ECD certification is reasonably straightforward but in the standard qualification, facilitators are not provided with disability-specific training, despite the fact that they may have children with disabilities in their care group. The Uhambo Foundation’s *Ndinogona* (“I Can”) programme is flexibly structured and suitable for use by carers, parents, and educators. The programme provides the means for disabled children to play (“structured stimulation”) and facilitates their integration into daily educational activities early, to ease the transition to schooling. The programme upskills adults to better include children with disabilities in educational settings. It is supported by a carefully prepared “stim kit”—toys that are specifically designed to be appealing and useful for children with a varied range of disabilities [64]. The programme also encourages educators and caregivers to interact more with children with disabilities and to be creative in their approach to disability inclusion. It tries to help communities and carers to recognise and act on the learning potential of children with disabilities (which may be displayed differently by these children in comparison to others [65]), thereby challenging existing stereotyped attitudes about disability. Finally, it recognises that all children, as well as their families and communities at large, can also benefit from this engagement and understanding of their peers with disabilities. The *Ndinogona* programme has a high likelihood of local acceptability and success, as these centres are already well-established in many communities. In this instance, the Uhambo Foundation observed an existing service, noticed that there were gaps in provision for children with disabilities, and provided a specific enhancement to a service that already exists in order to expand the inclusivity and impact of this important service.

A third example of the work that SSE conducts is their support and resourcing of caregivers. One of the Uhambo Foundation’s goals is to empower and teach caregivers and educators of children with disabilities by equipping them to navigate their own and their children’s challenges, and capacitating them to be advocates for disability rights and service provision. Toward these goals, the Champions of Change Trust was recently launched. This is a network of families who will connect with, educate, and conduct outreach with others who have similar experiences, as well as link them to important services, resources, and information. The organisation hopes this initiative will help to address and decrease the physical, emotional, and experiential isolation many parents of children with disabilities feel [19,66,67]. Focusing all its energy and attention on direct service provision can limit an organisation’s scale and sustainability. This kind of snowball capacitating approach works to empower caregivers and others involved in providing support, to reach as many people as possible, and to increase society’s willingness to support and include children and adults with disabilities.

## 3. Discussion

Various best practices and key recommendations from the broader literature on access to and the improved impact of assistive devices for people with disabilities are represented in the work of the Shonaquip Social Enterprise, as described above. It is important that these assistive devices be reviewed regularly to ensure needs are met across a growing child’s lifespan, and to ensure they are used as intended [68]. Policies that stipulate clear guidelines for the proper and equitable prescription and distribution of these devices are an important first step. However, it is also clear that the *impact* of these assistive devices can be massively enhanced by wider efforts to address the environmental and systemic exclusion that limits disabled children’s social participation. Toward shifting these broader constraining factors, the SSE collaborates with existing community networks and resources to strengthen referral pathways and optimise access to existing resources. They undergird these efforts by creating partnerships across the government departments of Health, Education, and Social Development, as well as by putting pressure on decision-makers to adapt or create suitable policy. They also train and support parents and caregivers as key advocates for disability and inclusion in the community. This broad-based stakeholder inclusivity speaks to the fact that everyone in a community has a role to play in driving disability inclusion.

A number of key lessons can be drawn from the example of the SSE. These include:

1. *Improving access to responsive, adaptive, and high-quality device design*: Assistive devices ought to be designed with the specific needs of clients and carers in mind, as well as a thorough understanding of local conditions, terrains, and environments [55,69]. A device that works well in a paved, urban environment with consistent ramp access may be totally inadequate for a child living in the rural and under-served urban environments that are far more common in South Africa and LMICs in general [62]. In other words, context matters. The importance of context-appropriateness is clearly demonstrated in the example of the Madiba2GoBuggy, which was designed with specific local circumstances in mind. Additional important factors that should be considered when assessing the utility of assistive devices include affordability, whether the device can be serviced or maintained in the most remote areas, and if it is possible to access regular reviews by rehabilitation or seating professionals [70]. Feedback from carers and users on the ground informs any new designs. For children, it is also necessary to consider caregiver feedback, which is often overlooked, and whether the device is still meeting the needs of a growing child [65,71].
2. *Training, capacitating the workforce, and upskilling human resources for disability service provision*: Devices alone will not improve inclusion—a better trained workforce is important. This training ought to be aligned with internationally recommended best practice and WHO standards, but should also be assessed for suitability to or adapted for the local context. In this regard, the SSE provides ongoing mentoring and remote support, caregiver training, assessment training, and maintenance support to build capacity that is still severely lacking. SSE also supports community-based mobile clinics and other outreach, as well as providing ECD facilitator and university-level training to sustainably build and entrench decentralised service provision and expertise. These efforts are informed by regular contact in the field with families, carers, and service providers of children with disabilities, global guidelines, and ongoing participation in international communities of practise, such as the International Society of Wheelchair Professionals [72].
3. *Holistic efforts to support participation and advocate for rights*: A community-based approach is important in most settings, but especially in LMICs where accessing centralised services may be more costly or difficult. Building such approaches relies on fostering deep, trusted local relationships. High quality services are crucial but need to be embedded in empowerment initiatives and within sustainable systems. To this end, the SSE focuses on strengthening referral systems, network-building, community dialogues, and building common understanding between stakeholders to leverage opportunities for impact and inclusion.
4. *“Nothing about us without us”*: The slogan used by many social movements including the disability rights movement, SSE works hard to embody this goal by championing the voices of children with disabilities and their caregivers. A large proportion of their workforce is also disabled, which demonstrates the capacity of people with disabilities to contribute economically when an environment is inclusive. A consultative service assists other companies and organisations in improving employment equity for people with disabilities, an effective and applied form of advocacy. Another core aspect of the SSE’s work is their strong and active parent/primary caregiver network. The SSE is increasing and accelerating the involvement of these important partners in decision-making about support needs, current and future priorities, and research goals. An additional result of this approach is that parents who are impoverished and marginalised can gain confidence and skills and potentially become more economically active.
5. *Working to enhance rather than replace existing services*: From almost thirty years of working with government, the SSE has gained experience and useful lessons for effective collaboration. Some of these may be most relevant for South Africa but may also offer insights for others interested in these kinds of collaborations. SSE has long established relationships with key staff in the Departments of Health, Education, and Social Development. Forming strong and consistent relationships at all levels is critical. Just as important is deep immersion at the site of intervention, in order to properly understand and include key local players. Part of this process involves ensuring that contractual agreements are in place, and that these include a detailed allocation of relative responsibilities and targets [73]. Strong reporting systems are also important [73], and the SSE uses an impact framework to improve accountability and report on any funded projects. Government partners are considered “joint service providers” in that they work with but do not see SSE as external expert service providers. The ultimate aim is to build capacity and offer mentoring for local service providers (both in and beyond government) so that the relevant service is long-term sustainable.
6. *Advocacy at all levels: The SSE is involved in lobbying and advocacy work at a range of levels*: from the local to the national, and across a number of government departments including the Departments of Health, Education, and Social Development. It is important to the organisation that their grassroots partners’ knowledge is incorporated into and shared through this advocacy work. They also hope to empower parents to collectively challenge the lack of service delivery and decision-making aligned to the country’s stated policies to protect and uphold the rights of their and other children with disabilities.

## 4. Conclusions

Though access to high quality appropriate assistive devices is indispensable, both global conversations and the overall story of the SSE suggest that it is important to focus on a holistic approach that aims for the ultimate goal of inclusion and participation, rather than on the distribution of devices alone. In a global context with a great emphasis on technology and what it can achieve, it is important to design well and more universally, to try and enable increased participation. However, it is also necessary to return to fundamental lessons dating from the social model of disability and later adapted in the WHO’s International Classification of Functioning, Disability and Health (ICF) and related approaches [55,74]. Disability inclusion is not just about managing impairments. It is also about challenging structural and attitudinal barriers in the broader environment so as to effect community-led and practical movement toward inclusion, a goal which is valuable for both disabled and non-disabled people [75]. Resources also ought to be directed toward gathering strong evidence from around the world and identifying sustainable, contextually relevant approaches to improving the lives and inclusion of disabled people in the global south [76].

A well-designed device which meets the needs of the child in their context has great potential to be a tool for inclusion but does not on its own allow the child to reach their full potential. Participation can only be fully realised when the user and their device are supported by an environment of trained parents, carers, educators, and rehabilitation workers. Importantly, inclusive environments can only be created when communities understand and welcome all children with disabilities as integral, engaged members of their communities. The development of adequate, well-considered devices and products is critical but must be supported by policy, resources in the workforce, appropriate training, and integration with existing provisions by government and other actors [55,74]. This is likely to raise different challenges in different socio-political contexts.

The SSE is as interested in producing functional, user-friendly devices as it is in helping children develop optimally, supporting parents in their caring and in other aspects of their lives, conscientizing and enhancing the work of rehabilitation workers, and changing societal attitudes. Devices that work make people with disabilities more visible, allow them more opportunities to participate, and have a beneficial impact on physical health and emotional wellbeing. This is part of the cycle of inclusion where the key question becomes not how a device does or does not work, but how a society at all levels becomes a place in which *all* have the potential to maximally contribute and benefit. Embedding the provision of these devices in a community of networked and resourceful groups and individuals who work together is far more likely to facilitate the goal of improved inclusion and fuller social, educational, and community participation.

We do not claim that everything that the SSE does is unique. However, it is useful to reflect on the history and impact of this social enterprise, partly because it has been successful for many years and continues to sustainably expand its services and support. The SSE also demonstrates the importance of holistic, collaborative thinking and activism, which is always a work in progress. A substantial growth opportunity for the SSE is to research and evaluate its work more formally, a development endeavour with which the SSE is currently actively engaged. Strategically, this kind of research is more likely to be acknowledged by powerful role-players and can be leveraged for advocacy and funding. In turn, this kind of collaboration enriches the evidence co-created by academic partners [74]. It is important that the local evidence base grows and is used to support and put pressure on government to deliver thoughtfully and comprehensively on its stated commitments [55]. We emphasise that we do not have the rigorous evaluation data we would wish for at this stage, and we would welcome debate about the key drivers for inclusion that we have suggested. We would also welcome inputs on how best to evaluate a complex set of activities and interventions when the ultimate goal (disability inclusion and participation) is deeply affected by factors outside the control and influence of service and empowerment organisations.
